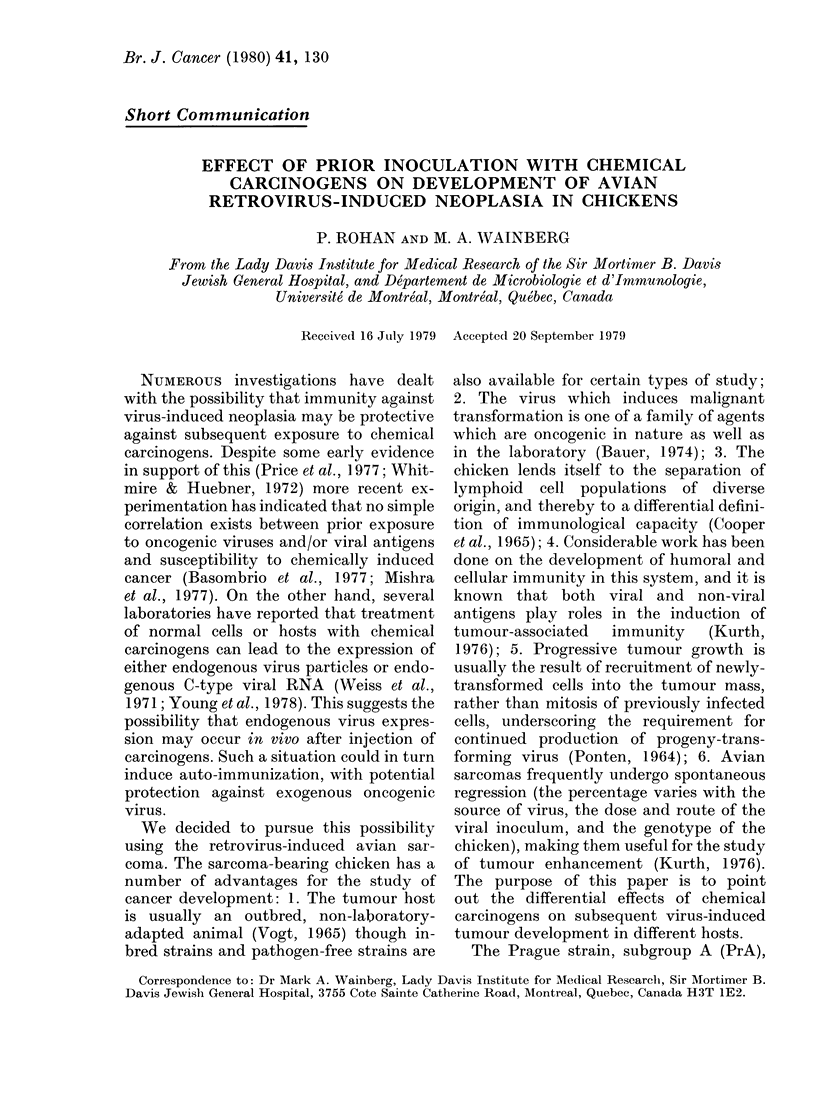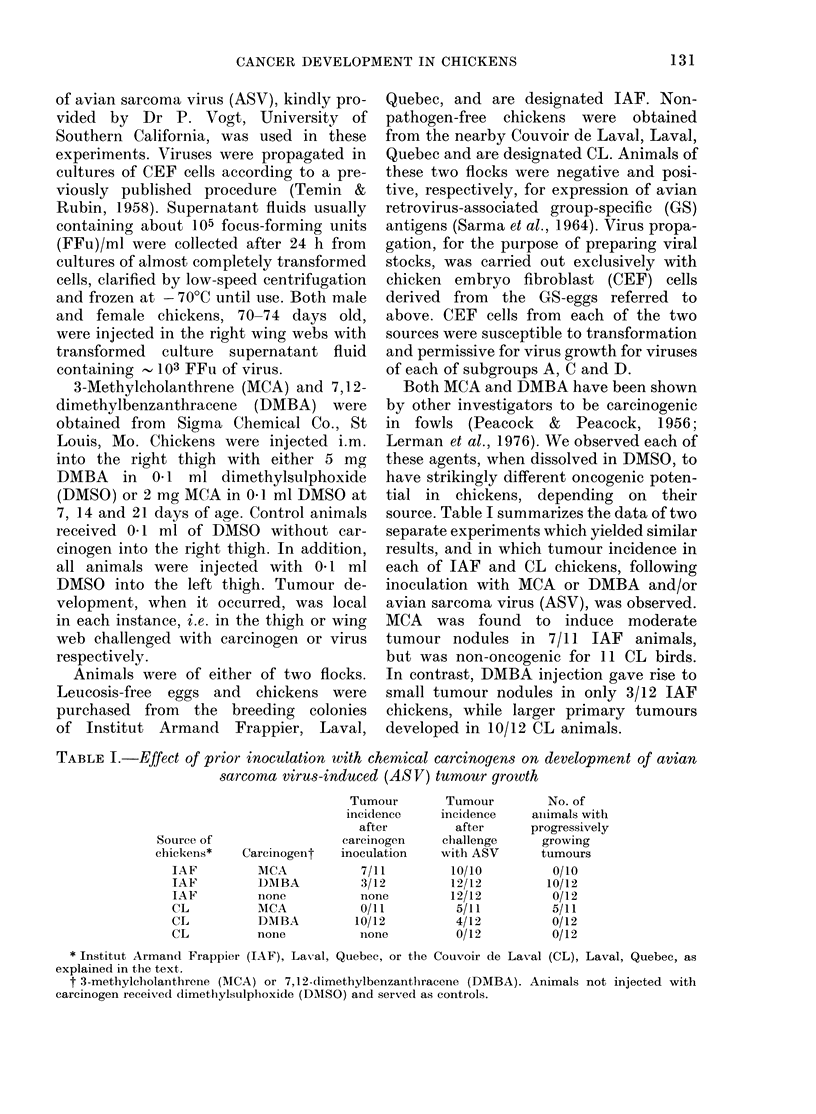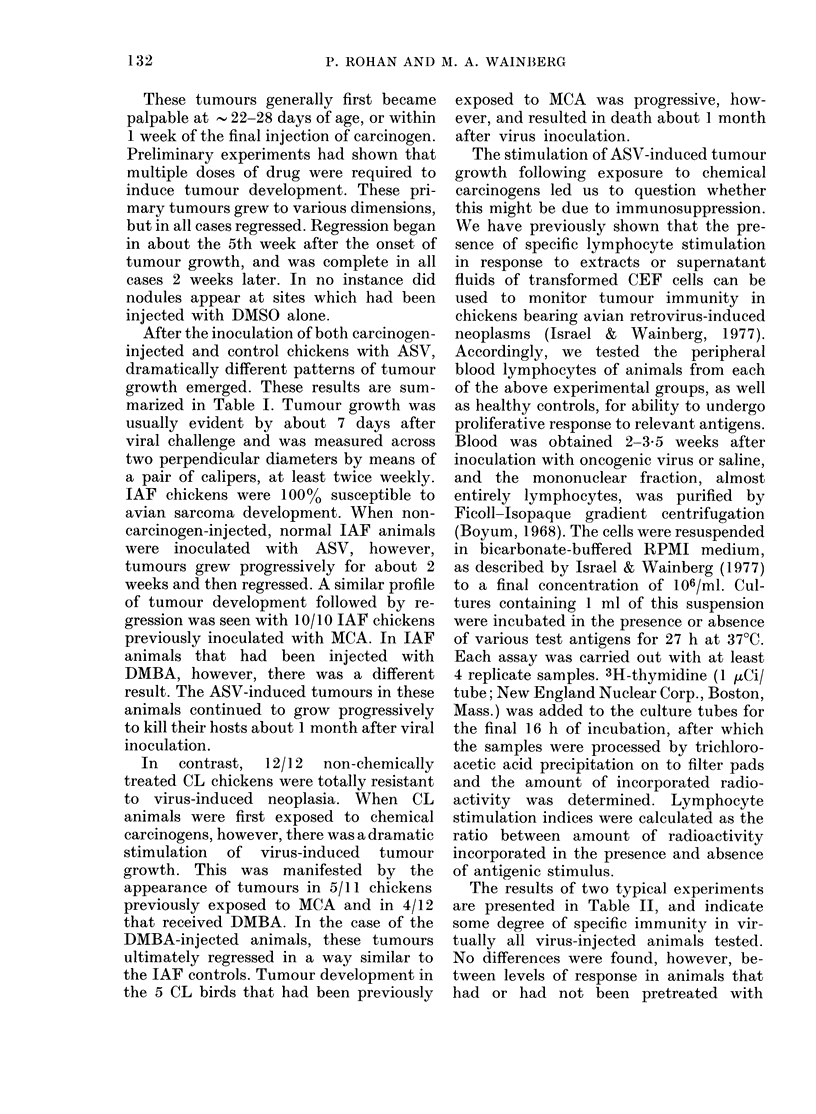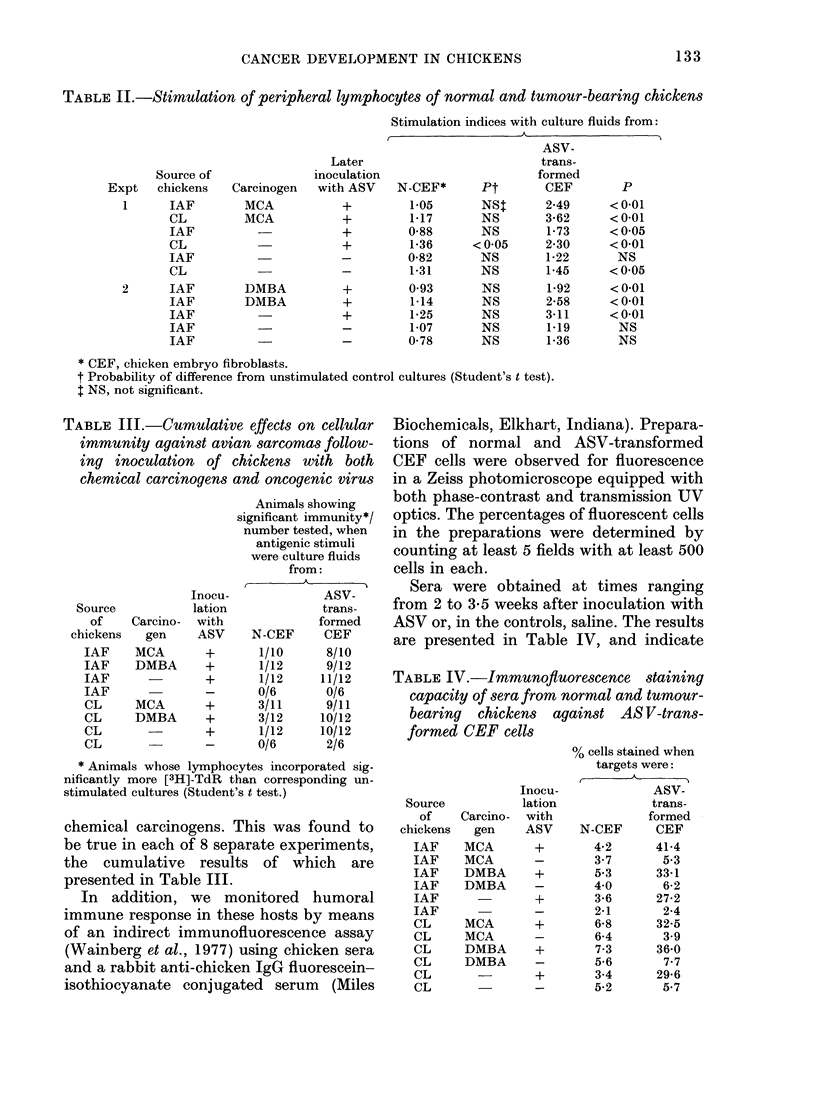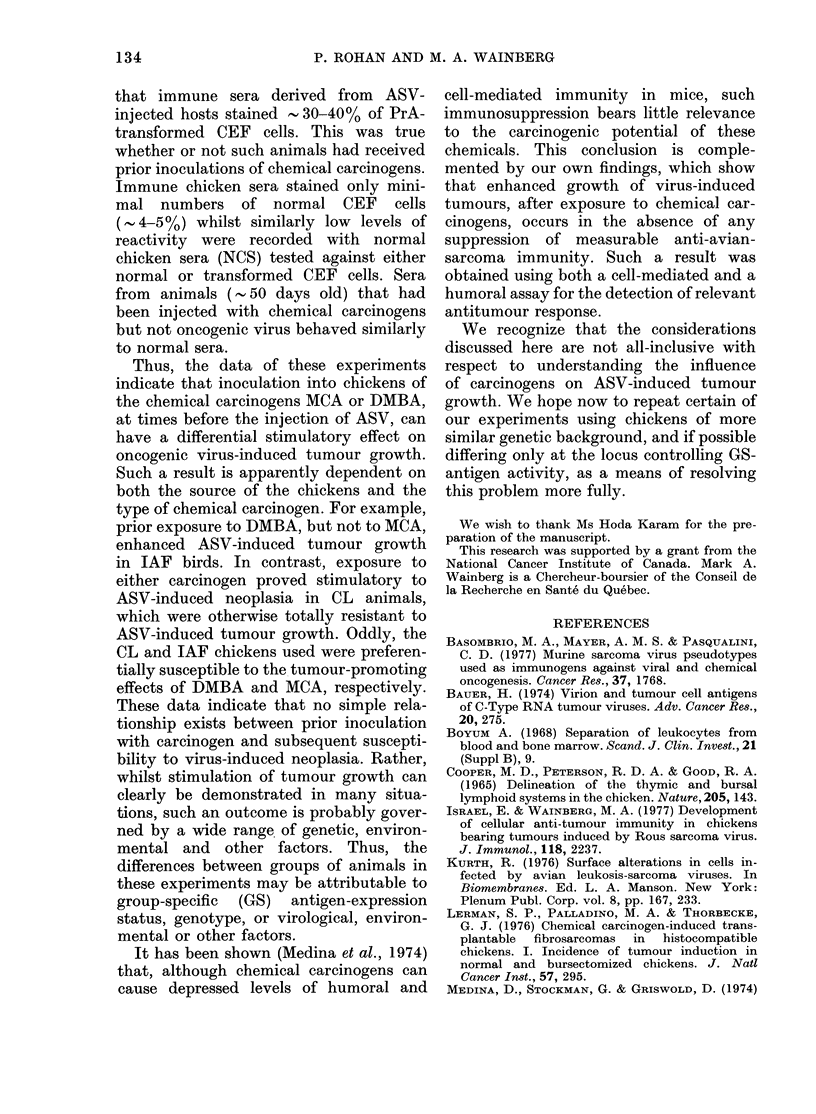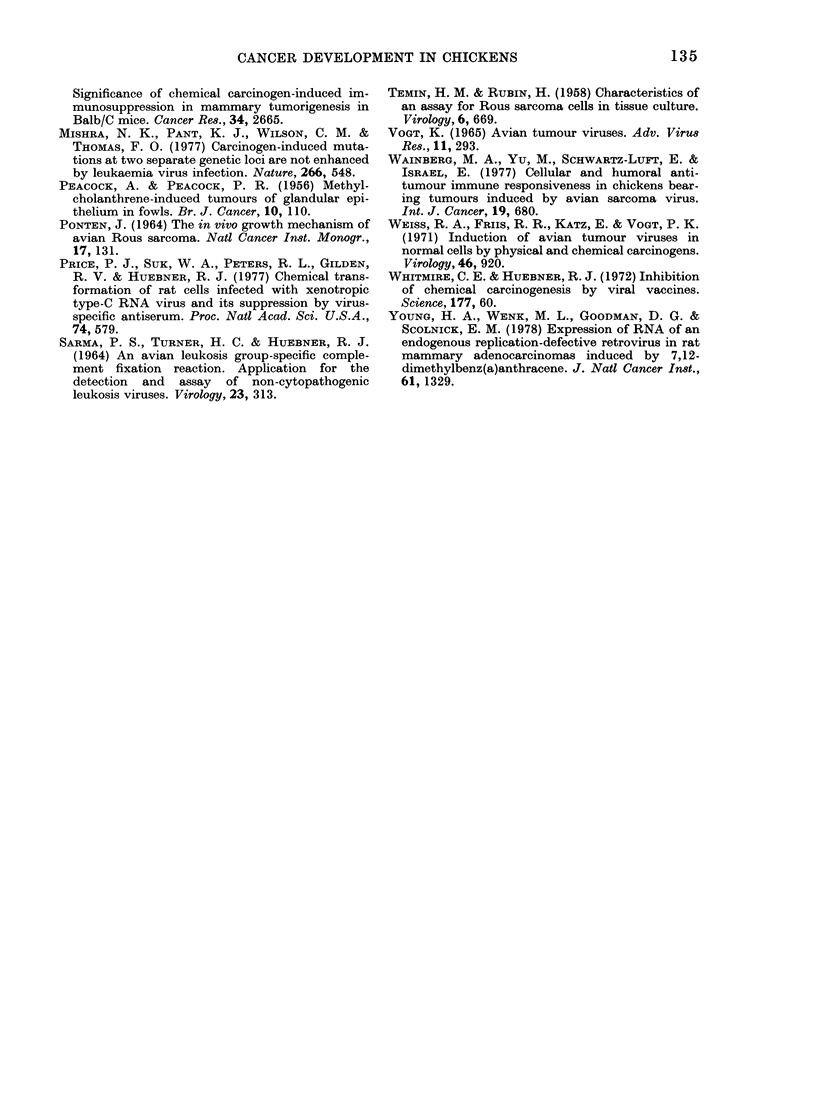# Effect of prior inoculation with chemical carcinogens on development of avian retrovirus-induced neoplasia in chickens.

**DOI:** 10.1038/bjc.1980.15

**Published:** 1980-01

**Authors:** P. Rohan, M. A. Wainberg


*Br. J. Cancer* (1980) **41**, 130

***Short Communication***

### EFFECT OF PRIOR INOCULATION WITH CHEMICAL CARCINOGENS ON DEVELOPMENT OF AVIAN RETROVIRUS-INDUCED NEOPLASIA IN CHICKENS

P. ROHAN AND M. A. WAINBERG

*From the Lady Davis Institute for Medical Research of the Sir Mortimer B. Davis Jewish General Hospital, and Département de Microbiologie et d'Immunologie, Université de Montréal, Montréal, Québec, Canada*

Received 16 July 1979 Accepted 20 September 1979

NUMEROUS investigations have dealt with the possibility that immunity against virus-induced neoplasia may be protective against subsequent exposure to chemical carcinogens. Despite some early evidence in support of this (Price *et al.*, 1977; Whitmire & Huebner, 1972) more recent experimentation has indicated that no simple correlation exists between prior exposure to oncogenic viruses and/or viral antigens and susceptibility to chemically induced cancer (Basombrio *et al.*, 1977; Mishra *et al.*, 1977). On the other hand, several laboratories have reported that treatment of normal cells or hosts with chemical carcinogens can lead to the expression of either endogenous virus particles or endogenous C-type viral RNA (Weiss *et al.*, 1971; Young *et al.*, 1978). This suggests the possibility that endogenous virus expression may occur *in vivo* after injection of carcinogens. Such a situation could in turn induce auto-immunization, with potential protection against exogenous oncogenic virus.

We decided to pursue this possibility using the retrovirus-induced avian sarcoma. The sarcoma-bearing chicken has a number of advantages for the study of cancer development: 1. The tumour host is usually an outbred, non-laboratory-adapted animal (Vogt, 1965) though inbred strains and pathogen-free strains are also available for certain types of study; 2. The virus which induces malignant transformation is one of a family of agents which are oncogenic in nature as well as in the laboratory (Bauer, 1974); 3. The chicken lends itself to the separation of lymphoid cell populations of diverse origin, and thereby to a differential definition of immunological capacity (Cooper *et al.*, 1965); 4. Considerable work has been done on the development of humoral and cellular immunity in this system, and it is known that both viral and non-viral antigens play roles in the induction of tumour-associated immunity (Kurth, 1976); 5. Progressive tumour growth is usually the result of recruitment of newly-transformed cells into the tumour mass, rather than mitosis of previously infected cells, underscoring the requirement for continued production of progeny-transforming virus (Ponten, 1964); 6. Avian sarcomas frequently undergo spontaneous regression (the percentage varies with the source of virus, the dose and route of the viral inoculum, and the genotype of the chicken), making them useful for the study of tumour enhancement (Kurth, 1976). The purpose of this paper is to point out the differential effects of chemical carcinogens on subsequent virus-induced tumour development in different hosts.

The Prague strain, subgroup A (PrA),

Correspondence to: Dr Mark A. Wainberg, Lady Davis Institute for Medical Research, Sir Mortimer B. Davis Jewish General Hospital, 3755 Cote Sainte Catherine Road, Montreal, Quebec, Canada H3T 1E2.

of avian sarcoma virus (ASV), kindly provided by Dr P. Vogt, University of Southern California, was used in these experiments. Viruses were propagated in cultures of CEF cells according to a previously published procedure (Temin & Rubin, 1958). Supernatant fluids usually containing about $10^5$ focus-forming units (FFu)/ml were collected after 24 h from cultures of almost completely transformed cells, clarified by low-speed centrifugation and frozen at $-70°C$ until use. Both male and female chickens, 70–74 days old, were injected in the right wing webs with transformed culture supernatant fluid containing $\sim 10^3$ FFu of virus.

3-Methylcholanthrene (MCA) and 7,12-dimethylbenzanthracene (DMBA) were obtained from Sigma Chemical Co., St Louis, Mo. Chickens were injected i.m. into the right thigh with either 5 mg DMBA in 0·1 ml dimethylsulphoxide (DMSO) or 2 mg MCA in 0·1 ml DMSO at 7, 14 and 21 days of age. Control animals received 0·1 ml of DMSO without carcinogen into the right thigh. In addition, all animals were injected with 0·1 ml DMSO into the left thigh. Tumour development, when it occurred, was local in each instance, *i.e.* in the thigh or wing web challenged with carcinogen or virus respectively.

Animals were of either of two flocks. Leucosis-free eggs and chickens were purchased from the breeding colonies of Institut Armand Frappier, Laval, Quebec, and are designated IAF. Non-pathogen-free chickens were obtained from the nearby Couvoir de Laval, Laval, Quebec and are designated CL. Animals of these two flocks were negative and positive, respectively, for expression of avian retrovirus-associated group-specific (GS) antigens (Sarma *et al.*, 1964). Virus propagation, for the purpose of preparing viral stocks, was carried out exclusively with chicken embryo fibroblast (CEF) cells derived from the GS-eggs referred to above. CEF cells from each of the two sources were susceptible to transformation and permissive for virus growth for viruses of each of subgroups A, C and D.

Both MCA and DMBA have been shown by other investigators to be carcinogenic in fowls (Peacock & Peacock, 1956; Lerman *et al.*, 1976). We observed each of these agents, when dissolved in DMSO, to have strikingly different oncogenic potential in chickens, depending on their source. Table I summarizes the data of two separate experiments which yielded similar results, and in which tumour incidence in each of IAF and CL chickens, following inoculation with MCA or DMBA and/or avian sarcoma virus (ASV), was observed. MCA was found to induce moderate tumour nodules in 7/11 IAF animals, but was non-oncogenic for 11 CL birds. In contrast, DMBA injection gave rise to small tumour nodules in only 3/12 IAF chickens, while larger primary tumours developed in 10/12 CL animals.

TABLE I.—*Effect of prior inoculation with chemical carcinogens on development of avian sarcoma virus-induced (ASV) tumour growth*

| Source of chickens* | Carcinogen† | Tumour incidence after carcinogen inoculation | Tumour incidence after challenge with ASV | No. of animals with progressively growing tumours |
|---|---|---|---|---|
| IAF | MCA | 7/11 | 10/10 | 0/10 |
| IAF | DMBA | 3/12 | 12/12 | 10/12 |
| IAF | none | none | 12/12 | 0/12 |
| CL | MCA | 0/11 | 5/11 | 5/11 |
| CL | DMBA | 10/12 | 4/12 | 0/12 |
| CL | none | none | 0/12 | 0/12 |

* Institut Armand Frappier (IAF), Laval, Quebec, or the Couvoir de Laval (CL), Laval, Quebec, as explained in the text.

† 3-methylcholanthrene (MCA) or 7,12-dimethylbenzanthracene (DMBA). Animals not injected with carcinogen received dimethylsulphoxide (DMSO) and served as controls.

These tumours generally first became palpable at ~22–28 days of age, or within 1 week of the final injection of carcinogen. Preliminary experiments had shown that multiple doses of drug were required to induce tumour development. These primary tumours grew to various dimensions, but in all cases regressed. Regression began in about the 5th week after the onset of tumour growth, and was complete in all cases 2 weeks later. In no instance did nodules appear at sites which had been injected with DMSO alone.

After the inoculation of both carcinogen-injected and control chickens with ASV, dramatically different patterns of tumour growth emerged. These results are summarized in Table I. Tumour growth was usually evident by about 7 days after viral challenge and was measured across two perpendicular diameters by means of a pair of calipers, at least twice weekly. IAF chickens were 100% susceptible to avian sarcoma development. When non-carcinogen-injected, normal IAF animals were inoculated with ASV, however, tumours grew progressively for about 2 weeks and then regressed. A similar profile of tumour development followed by regression was seen with 10/10 IAF chickens previously inoculated with MCA. In IAF animals that had been injected with DMBA, however, there was a different result. The ASV-induced tumours in these animals continued to grow progressively to kill their hosts about 1 month after viral inoculation.

In contrast, 12/12 non-chemically treated CL chickens were totally resistant to virus-induced neoplasia. When CL animals were first exposed to chemical carcinogens, however, there was a dramatic stimulation of virus-induced tumour growth. This was manifested by the appearance of tumours in 5/11 chickens previously exposed to MCA and in 4/12 that received DMBA. In the case of the DMBA-injected animals, these tumours ultimately regressed in a way similar to the IAF controls. Tumour development in the 5 CL birds that had been previously exposed to MCA was progressive, however, and resulted in death about 1 month after virus inoculation.

The stimulation of ASV-induced tumour growth following exposure to chemical carcinogens led us to question whether this might be due to immunosuppression. We have previously shown that the presence of specific lymphocyte stimulation in response to extracts or supernatant fluids of transformed CEF cells can be used to monitor tumour immunity in chickens bearing avian retrovirus-induced neoplasms (Israel & Wainberg, 1977). Accordingly, we tested the peripheral blood lymphocytes of animals from each of the above experimental groups, as well as healthy controls, for ability to undergo proliferative response to relevant antigens. Blood was obtained 2–3·5 weeks after inoculation with oncogenic virus or saline, and the mononuclear fraction, almost entirely lymphocytes, was purified by Ficoll–Isopaque gradient centrifugation (Boyum, 1968). The cells were resuspended in bicarbonate-buffered RPMI medium, as described by Israel & Wainberg (1977) to a final concentration of $10^6$/ml. Cultures containing 1 ml of this suspension were incubated in the presence or absence of various test antigens for 27 h at 37°C. Each assay was carried out with at least 4 replicate samples. $^3$H-thymidine (1 $\mu$Ci/tube; New England Nuclear Corp., Boston, Mass.) was added to the culture tubes for the final 16 h of incubation, after which the samples were processed by trichloroacetic acid precipitation on to filter pads and the amount of incorporated radioactivity was determined. Lymphocyte stimulation indices were calculated as the ratio between amount of radioactivity incorporated in the presence and absence of antigenic stimulus.

The results of two typical experiments are presented in Table II, and indicate some degree of specific immunity in virtually all virus-injected animals tested. No differences were found, however, between levels of response in animals that had or had not been pretreated with

TABLE II.—*Stimulation of peripheral lymphocytes of normal and tumour-bearing chickens*

| | | | | Stimulation indices with culture fluids from: | | | |
|---|---|---|---|---|---|---|---|
| Expt | Source of chickens | Carcinogen | Later inoculation with ASV | N-CEF* | *P*† | ASV-transformed CEF | *P* |
| 1 | IAF | MCA | + | 1·05 | NS‡ | 2·49 | <0·01 |
| | CL | MCA | + | 1·17 | NS | 3·62 | <0·01 |
| | IAF | — | + | 0·88 | NS | 1·73 | <0·05 |
| | CL | — | + | 1·36 | <0·05 | 2·30 | <0·01 |
| | IAF | — | – | 0·82 | NS | 1·22 | NS |
| | CL | — | – | 1·31 | NS | 1·45 | <0·05 |
| 2 | IAF | DMBA | + | 0·93 | NS | 1·92 | <0·01 |
| | IAF | DMBA | + | 1·14 | NS | 2·58 | <0·01 |
| | IAF | — | + | 1·25 | NS | 3·11 | <0·01 |
| | IAF | — | – | 1·07 | NS | 1·19 | NS |
| | IAF | — | – | 0·78 | NS | 1·36 | NS |

* CEF, chicken embryo fibroblasts.
† Probability of difference from unstimulated control cultures (Student's *t* test).
‡ NS, not significant.

TABLE III.—*Cumulative effects on cellular immunity against avian sarcomas following inoculation of chickens with both chemical carcinogens and oncogenic virus*

| | | | Animals showing significant immunity*/number tested, when antigenic stimuli were culture fluids from: | |
|---|---|---|---|---|
| Source of chickens | Carcinogen | Inoculation with ASV | N-CEF | ASV-transformed CEF |
| IAF | MCA | + | 1/10 | 8/10 |
| IAF | DMBA | + | 1/12 | 9/12 |
| IAF | — | + | 1/12 | 11/12 |
| IAF | — | – | 0/6 | 0/6 |
| CL | MCA | + | 3/11 | 9/11 |
| CL | DMBA | + | 3/12 | 10/12 |
| CL | — | + | 1/12 | 10/12 |
| CL | — | – | 0/6 | 2/6 |

* Animals whose lymphocytes incorporated significantly more [$^3$H]-TdR than corresponding unstimulated cultures (Student's *t* test.)

chemical carcinogens. This was found to be true in each of 8 separate experiments, the cumulative results of which are presented in Table III.

In addition, we monitored humoral immune response in these hosts by means of an indirect immunofluorescence assay (Wainberg *et al.*, 1977) using chicken sera and a rabbit anti-chicken IgG fluorescein–isothiocyanate conjugated serum (Miles Biochemicals, Elkhart, Indiana). Preparations of normal and ASV-transformed CEF cells were observed for fluorescence in a Zeiss photomicroscope equipped with both phase-contrast and transmission UV optics. The percentages of fluorescent cells in the preparations were determined by counting at least 5 fields with at least 500 cells in each.

Sera were obtained at times ranging from 2 to 3·5 weeks after inoculation with ASV or, in the controls, saline. The results are presented in Table IV, and indicate

TABLE IV.—*Immunofluorescence staining capacity of sera from normal and tumour-bearing chickens against ASV-transformed CEF cells*

| | | | % cells stained when targets were: | |
|---|---|---|---|---|
| Source of chickens | Carcinogen | Inoculation with ASV | N-CEF | ASV-transformed CEF |
| IAF | MCA | + | 4·2 | 41·4 |
| IAF | MCA | – | 3·7 | 5·3 |
| IAF | DMBA | + | 5·3 | 33·1 |
| IAF | DMBA | – | 4·0 | 6·2 |
| IAF | — | + | 3·6 | 27·2 |
| IAF | — | – | 2·1 | 2·4 |
| CL | MCA | + | 6·8 | 32·5 |
| CL | MCA | – | 6·4 | 3·9 |
| CL | DMBA | + | 7·3 | 36·0 |
| CL | DMBA | – | 5·6 | 7·7 |
| CL | — | + | 3·4 | 29·6 |
| CL | — | – | 5·2 | 5·7 |

that immune sera derived from ASV-injected hosts stained ~30–40% of PrA-transformed CEF cells. This was true whether or not such animals had received prior inoculations of chemical carcinogens. Immune chicken sera stained only minimal numbers of normal CEF cells (~4–5%) whilst similarly low levels of reactivity were recorded with normal chicken sera (NCS) tested against either normal or transformed CEF cells. Sera from animals (~50 days old) that had been injected with chemical carcinogens but not oncogenic virus behaved similarly to normal sera.

Thus, the data of these experiments indicate that inoculation into chickens of the chemical carcinogens MCA or DMBA, at times before the injection of ASV, can have a differential stimulatory effect on oncogenic virus-induced tumour growth. Such a result is apparently dependent on both the source of the chickens and the type of chemical carcinogen. For example, prior exposure to DMBA, but not to MCA, enhanced ASV-induced tumour growth in IAF birds. In contrast, exposure to either carcinogen proved stimulatory to ASV-induced neoplasia in CL animals, which were otherwise totally resistant to ASV-induced tumour growth. Oddly, the CL and IAF chickens used were preferentially susceptible to the tumour-promoting effects of DMBA and MCA, respectively. These data indicate that no simple relationship exists between prior inoculation with carcinogen and subsequent susceptibility to virus-induced neoplasia. Rather, whilst stimulation of tumour growth can clearly be demonstrated in many situations, such an outcome is probably governed by a wide range of genetic, environmental and other factors. Thus, the differences between groups of animals in these experiments may be attributable to group-specific (GS) antigen-expression status, genotype, or virological, environmental or other factors.

It has been shown (Medina *et al.*, 1974) that, although chemical carcinogens can cause depressed levels of humoral and cell-mediated immunity in mice, such immunosuppression bears little relevance to the carcinogenic potential of these chemicals. This conclusion is complemented by our own findings, which show that enhanced growth of virus-induced tumours, after exposure to chemical carcinogens, occurs in the absence of any suppression of measurable anti-avian-sarcoma immunity. Such a result was obtained using both a cell-mediated and a humoral assay for the detection of relevant antitumour response.

We recognize that the considerations discussed here are not all-inclusive with respect to understanding the influence of carcinogens on ASV-induced tumour growth. We hope now to repeat certain of our experiments using chickens of more similar genetic background, and if possible differing only at the locus controlling GS-antigen activity, as a means of resolving this problem more fully.

We wish to thank Ms Hoda Karam for the preparation of the manuscript.

This research was supported by a grant from the National Cancer Institute of Canada. Mark A. Wainberg is a Chercheur-boursier of the Conseil de la Recherche en Santé du Québec.

### REFERENCES

BASOMBRIO, M. A., MAYER, A. M. S. & PASQUALINI, C. D. (1977) Murine sarcoma virus pseudotypes used as immunogens against viral and chemical oncogenesis. *Cancer Res.*, **37**, 1768.

BAUER, H. (1974) Virion and tumour cell antigens of C-Type RNA tumour viruses. *Adv. Cancer Res.*, **20**, 275.

BOYUM A. (1968) Separation of leukocytes from blood and bone marrow. *Scand. J. Clin. Invest.*, **21** (Suppl B), 9.

COOPER, M. D., PETERSON, R. D. A. & GOOD, R. A. (1965) Delineation of the thymic and bursal lymphoid systems in the chicken. *Nature*, **205**, 143.

ISRAEL, E. & WAINBERG, M. A. (1977) Development of cellular anti-tumour immunity in chickens bearing tumours induced by Rous sarcoma virus. *J. Immunol.*, **118**, 2237.

KURTH, R. (1976) Surface alterations in cells infected by avian leukosis-sarcoma viruses. In *Biomembranes*. Ed. L. A. Manson. New York: Plenum Publ. Corp. vol. 8, pp. 167, 233.

LERMAN, S. P., PALLADINO, M. A. & THORBECKE, G. J. (1976) Chemical carcinogen-induced transplantable fibrosarcomas in histocompatible chickens. I. Incidence of tumour induction in normal and bursectomized chickens. *J. Natl Cancer Inst.*, **57**, 295.

MEDINA, D., STOCKMAN, G. & GRISWOLD, D. (1974)

Significance of chemical carcinogen-induced immunosuppression in mammary tumorigenesis in Balb/C mice. *Cancer Res.*, **34**, 2665.

MISHRA, N. K., PANT, K. J., WILSON, C. M. & THOMAS, F. O. (1977) Carcinogen-induced mutations at two separate genetic loci are not enhanced by leukaemia virus infection. *Nature*, **266**, 548.

PEACOCK, A. & PEACOCK, P. R. (1956) Methylcholanthrene-induced tumours of glandular epithelium in fowls. *Br. J. Cancer*, **10**, 110.

PONTEN, J. (1964) The *in vivo* growth mechanism of avian Rous sarcoma. *Natl Cancer Inst. Monogr.*, **17**, 131.

PRICE, P. J., SUK, W. A., PETERS, R. L., GILDEN, R. V. & HUEBNER, R. J. (1977) Chemical transformation of rat cells infected with xenotropic type-C RNA virus and its suppression by virus-specific antiserum. *Proc. Natl Acad. Sci. U.S.A.*, **74**, 579.

SARMA, P. S., TURNER, H. C. & HUEBNER, R. J. (1964) An avian leukosis group-specific complement fixation reaction. Application for the detection and assay of non-cytopathogenic leukosis viruses. *Virology*, **23**, 313.

TEMIN, H. M. & RUBIN, H. (1958) Characteristics of an assay for Rous sarcoma cells in tissue culture. *Virology*, **6**, 669.

VOGT, K. (1965) Avian tumour viruses. *Adv. Virus Res.*, **11**, 293.

WAINBERG, M. A., YU, M., SCHWARTZ-LUFT, E. & ISRAEL, E. (1977) Cellular and humoral antitumour immune responsiveness in chickens bearing tumours induced by avian sarcoma virus. *Int. J. Cancer*, **19**, 680.

WEISS, R. A., FRIIS, R. R., KATZ, E. & VOGT, P. K. (1971) Induction of avian tumour viruses in normal cells by physical and chemical carcinogens. *Virology*, **46**, 920.

WHITMIRE, C. E. & HUEBNER, R. J. (1972) Inhibition of chemical carcinogenesis by viral vaccines. *Science*, **177**, 60.

YOUNG, H. A., WENK, M. L., GOODMAN, D. G. & SCOLNICK, E. M. (1978) Expression of RNA of an endogenous replication-defective retrovirus in rat mammary adenocarcinomas induced by 7,12-dimethylbenz(a)anthracene. *J. Natl Cancer Inst.*, **61**, 1329.